# A Royal Flush: Carcinoid Heart Disease Complicated by Severe Tricuspid and Pulmonic Valve Regurgitation

**DOI:** 10.7759/cureus.86644

**Published:** 2025-06-24

**Authors:** Syed Rafay H Zaidi, Nina Appareddy, Wallacy Garcia, Raj Vashistha, Jenny K Lee, Faisal Mahfooz, Derar Albashaireh

**Affiliations:** 1 Internal Medicine, University of Colorado Health (UCHealth) Parkview Medical Center, Pueblo, USA; 2 Cardiovascular Medicine, University of Colorado Health (UCHealth) Parkview Medical Center, Pueblo, USA; 3 Cardiovascular Medicine, Pueblo Cardiology Associates, Pueblo, USA

**Keywords:** carcinoid heart disease, neuroendocrine tumors, pulmonary valve insufficiency, somatostatin analogs, tricuspid valve insufficiency

## Abstract

Carcinoid heart disease (CHD) is a severe complication of metastatic neuroendocrine tumors (NETs), leading to fibrotic degeneration of right-sided heart valves. A 38-year-old male presented with progressive dyspnea, fatigue, abdominal bloating, diarrhea, and facial flushing. Imaging revealed hepatic metastases and mesenteric lymphadenopathy, and biochemical markers confirmed a NET of likely gastrointestinal origin. Echocardiography showed torrential tricuspid regurgitation, severe pulmonary insufficiency, right ventricular dilation, and a patent foramen ovale (PFO). The patient was started on long-acting lanreotide for carcinoid syndrome and optimized on heart failure therapy. Due to severe valvular dysfunction, he underwent tricuspid and pulmonic valve replacement with bioprosthetic valves and PFO closure under perioperative octreotide infusion. The patient also underwent transthoracic liver debulking and ablation, small bowel resection, and mesenteric dissection. Postoperatively, he showed symptomatic improvement and remains under multidisciplinary surveillance. This case highlights the importance of early recognition, multidisciplinary management, and surgical intervention in CHD to optimize outcomes. Early initiation of somatostatin analog therapy, guideline-directed medical therapy for heart failure, and timely surgical intervention can significantly improve symptom burden and survival.

## Introduction

Carcinoid heart disease (CHD) is a rare but serious complication of metastatic neuroendocrine tumors (NETs) occurring due to excessive secretion of serotonin by metastatic tumors, leading to endocardial fibrosis and valvular dysfunction [1,2]. Right-sided heart failure can ensue, significantly impacting morbidity and mortality. The diverse clinical manifestations include dyspnea, fatigue, peripheral edema, and ascites, which can be mistaken for other cardiac or systemic conditions [3,4]. Given the potential for delayed diagnosis and progression to severe cardiac dysfunction, early recognition and multidisciplinary intervention are critical.

## Case presentation

A 38-year-old male presented to the emergency department with progressively worsening abdominal pain over the last five months, which acutely worsened over the past two days. He also reported progressive dyspnea, fatigue, abdominal bloating, facial flushing, night sweats, unintentional weight loss, and intermittent diarrhea and rectal bleeding. He had a II/VI holosystolic murmur best appreciated at the apex, mild bilateral lower extremity edema, and mild generalized abdominal tenderness.

Contrast-enhanced computed tomography (CT) of the abdomen and pelvis demonstrated multiple rim-enhancing hepatic masses, mildly enlarged mesenteric lymph nodes in the porta hepatis and upper mesentery, a necrotic mass in the central mesentery, and diffuse colonic mucosal thickening. Triple-phase CT of the abdomen demonstrated multiple heterogeneously enhancing liver masses and a 2.4 cm mesenteric mass or enlarged lymph node, suggestive of malignancy. A primary tumor was not identified on CT chest imaging. Esophagogastroduodenoscopy (EGD) demonstrated an irregular Z-line of the esophagus suspicious for Barrett's esophagus, multiple erosions in the lesser curvature of the stomach, and a pre-pyloric erosive nodule, which were biopsied. Colonoscopy (CSP) demonstrated a generally normal colon with mild irritation in the terminal ileum, which was biopsied. No clear source for malignancy was identified, and biopsies were negative.

**Figure 1:**
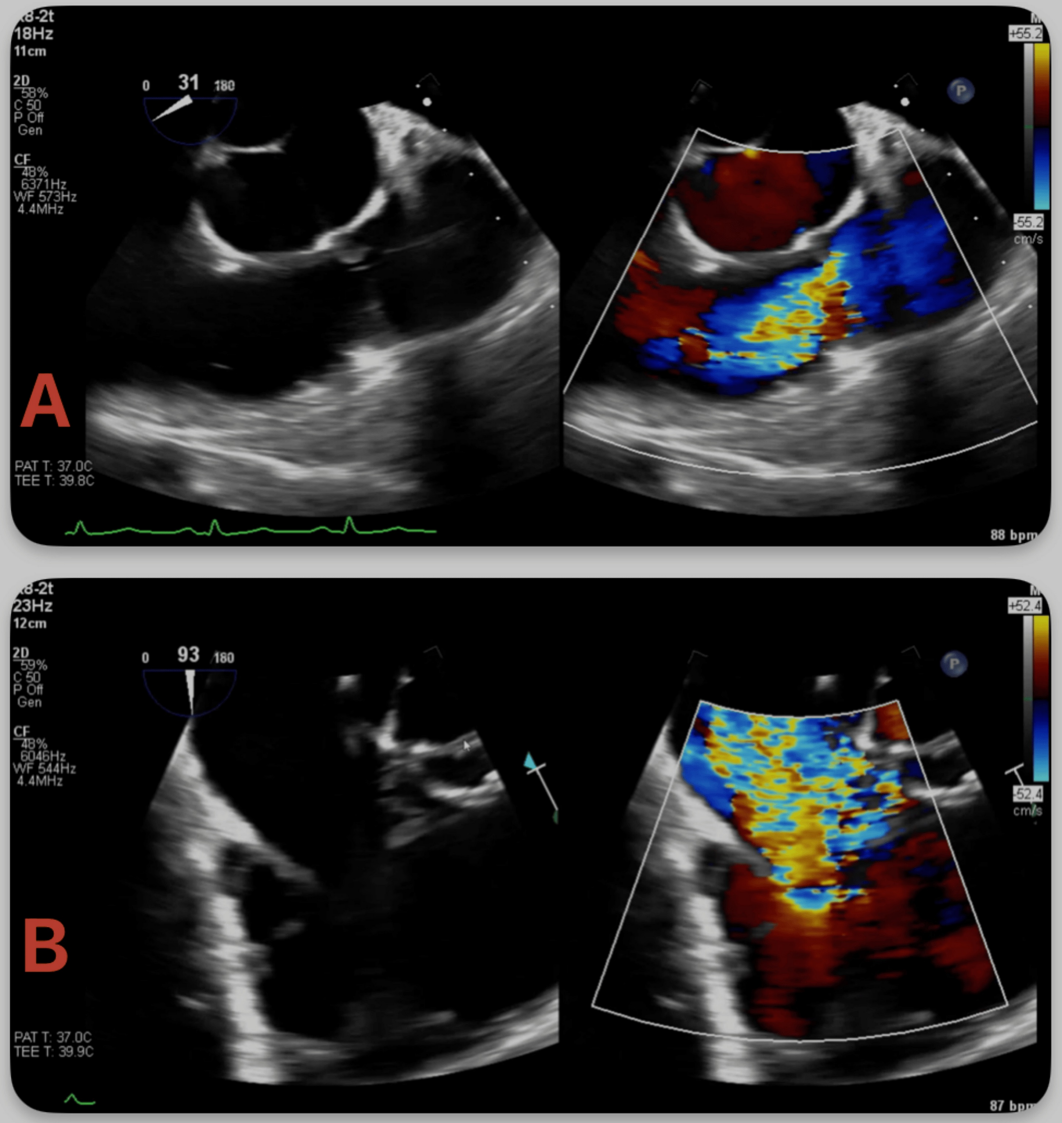
Transesophageal echocardiography (TEE) demonstrating carcinoid heart disease with dual right-sided valve involvement TEE demonstrating color compare Doppler imaging with thickened, retracted, and immobile leaflets with malcoaptation and resultant severe regurgitation of the (A) pulmonic and (B) tricuspid valves.

**Figure 2:**
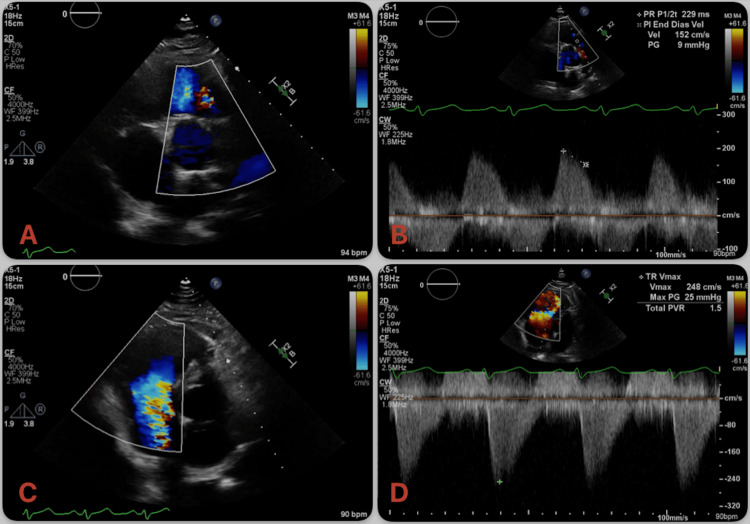
Transthoracic echocardiography (TTE) demonstrating carcinoid heart disease with dual right-sided valve involvement TTE demonstrating (A) severe pulmonic valve insufficiency (PI) in short-axis view, (B) with Doppler profile consistent with severe PI, and (C) restricted motion of the tricuspid leaflets with no coaptation and wide torrential tricuspid regurgitation, with (D) dagger-shaped Doppler profile, consistent with carcinoid heart disease.

Given the murmur noted on examination, a transthoracic echocardiogram (TTE) was performed and demonstrated severe pulmonic valve insufficiency and restricted motion of the tricuspid leaflets with no coaptation and wide torrential tricuspid regurgitation, raising suspicion of CHD (Figure 1A, 1B, Videos 1, 2). TTE furthermore demonstrated a severely dilated right atrium (RA) and right ventricle (RV) and flattening of the interventricular septum, suggestive of RV pressure and volume overload. TTE also demonstrated a patent foramen ovale (PFO) and a left ventricular ejection fraction of 35-40%. A transesophageal echocardiogram (TEE) was performed for further evaluation, which demonstrated thickened, retracted, and immobile tricuspid and pulmonic valve leaflets with malcoaptation and resultant severe regurgitation, consistent with CHD (Figure 2A-2D, Videos 3, 4). TEE further demonstrated dilated RA and RV with the interatrial septum bowing toward the left atrium, indicative of high right atrial pressure. The PFO appeared stretched, with color Doppler demonstrating a right-to-left interatrial shunt.

**Video 1:** Transthoracic echocardiography (TTE) demonstrating severe pulmonic valve insufficiency in short-axis view

**Video 2:** Transthoracic echocardiography (TTE) demonstrating restricted motion of the tricuspid leaflets with no coaptation and wide torrential tricuspid regurgitation

**Video 3:** Transesophageal echocardiography (TEE) color compare Doppler imaging with thickened, retracted, and immobile pulmonic valve leaflets with malcoaptation and resultant severe regurgitation

**Video 4:** Transesophageal echocardiography (TEE) color compare Doppler imaging with thickened, retracted, and immobile tricuspid valve leaflets with malcoaptation and resultant severe regurgitation

A CT-guided biopsy of the hepatic lesions showed a low-grade NET, likely metastatic, gastrointestinal grade 1, well-differentiated NET. A DOTATATE PET scan showed moderate hepatic disease involving the right and left hepatic lobes and mesenteric nodal metastatic disease with no obvious site of primary tumor. Laboratory evaluation demonstrated elevated chromogranin A and urinary 5-hydroxyindoleacetic acid (5-HIAA) levels, consistent with suspected neuroendocrine (Table 1).

**Table 1:** Tumor markers Results of tumor markers were obtained, demonstrating negative CEA, CA 19-9, and AFP levels and elevated chromogranin A and 5-HIAA levels.

Test	Result	Reference Range
Alpha-fetoprotein (AFP) tumor marker	1.8 ng/mL	0.0-6.9 ng/mL
Cancer antigen 19-9 (CA 19-9)	3.1 U/mL	0.0–37.0 U/mL
Carcinoembryonic antigen (CEA)	1.2 ng/mL	0-5 ng/mL
Chromogranin A	3136 ng/mL	0-187 ng/mL
24-hour 5-hydroxyindoleacetic acid (HIAA), urine ratio to creatinine excretion	175 mg/gCR	0-14 mg/gCR

Given the severity of valvular dysfunction with symptomatic right-sided heart failure, a multidisciplinary team approach was pursued, including cardiology, cardiothoracic surgery, hematology-oncology, and surgical oncology.

The patient was optimized on guideline-directed medical therapies for heart failure. He was also initiated on long-acting somatostatin analog therapy with lanreotide 120 mg every four weeks to reduce tumor secretion and mitigate carcinoid symptoms. He was offered and agreed upon surgical replacement of the tricuspid and pulmonic valves. Preoperative left and right heart catheterization demonstrated no coronary disease, a mean right atrial pressure of 11 mmHg with prominent v waves up to 23 mmHg consistent with severe tricuspid regurgitation, and normal pulmonary capillary wedge pressures, cardiac output, and cardiac index (Table 2). He received perioperative octreotide infusion to mitigate carcinoid crisis and underwent surgical PFO closure and tricuspid (31 mm Epic™ bioprosthetic valve, Abbott, IL, USA) and pulmonic valve replacement (29 mm Edwards Magna tissue valve, Edwards Lifesciences, CA, USA). Tissue valves were selected due to the anticipated need for future oncologic surgeries requiring interruption of anticoagulation.

**Table 2:** Right heart catheterization (RHC) RHC demonstrating mean right atrial pressure of 11 mmHg with prominent v waves up to 23 mmHg, consistent with severe tricuspid regurgitation. Normal pulmonary capillary wedge pressures, cardiac output, and cardiac index were noted.

Parameters	Measurements
Right atrial pressure	11 mmHg prominent v waves up to 23 mmHg
Right ventricular pressure (systolic, diastolic)	28, 11 mmHg
Pulmonary arterial pressure ((systolic/diastolic (mean))	21/11 (13) mmHg
Pulmonary capillary wedge pressure	13 mmHg
Cardiac output (Thermodilution)	4.53
Cardiac output (Fick)	4.05
Cardiac index (Thermodilution)	2.31
Cardiac index (Fick)	2.17
Pulmonary artery saturation	67%
Arterial oxygen saturation	94% on room air

Following completion of three months of postoperative warfarin therapy, the patient underwent transthoracic liver debulking and liver tumor ablation, small bowel resection, and mesenteric dissection with surgical oncology. Surgical pathology showed a well-differentiated NET, grade 1, strongly and diffusely positive for somatostatin type II receptor. Findings were ultimately consistent with a diagnosis of metastatic small intestinal NET with CHD, stage IV.

**Figure 3:**
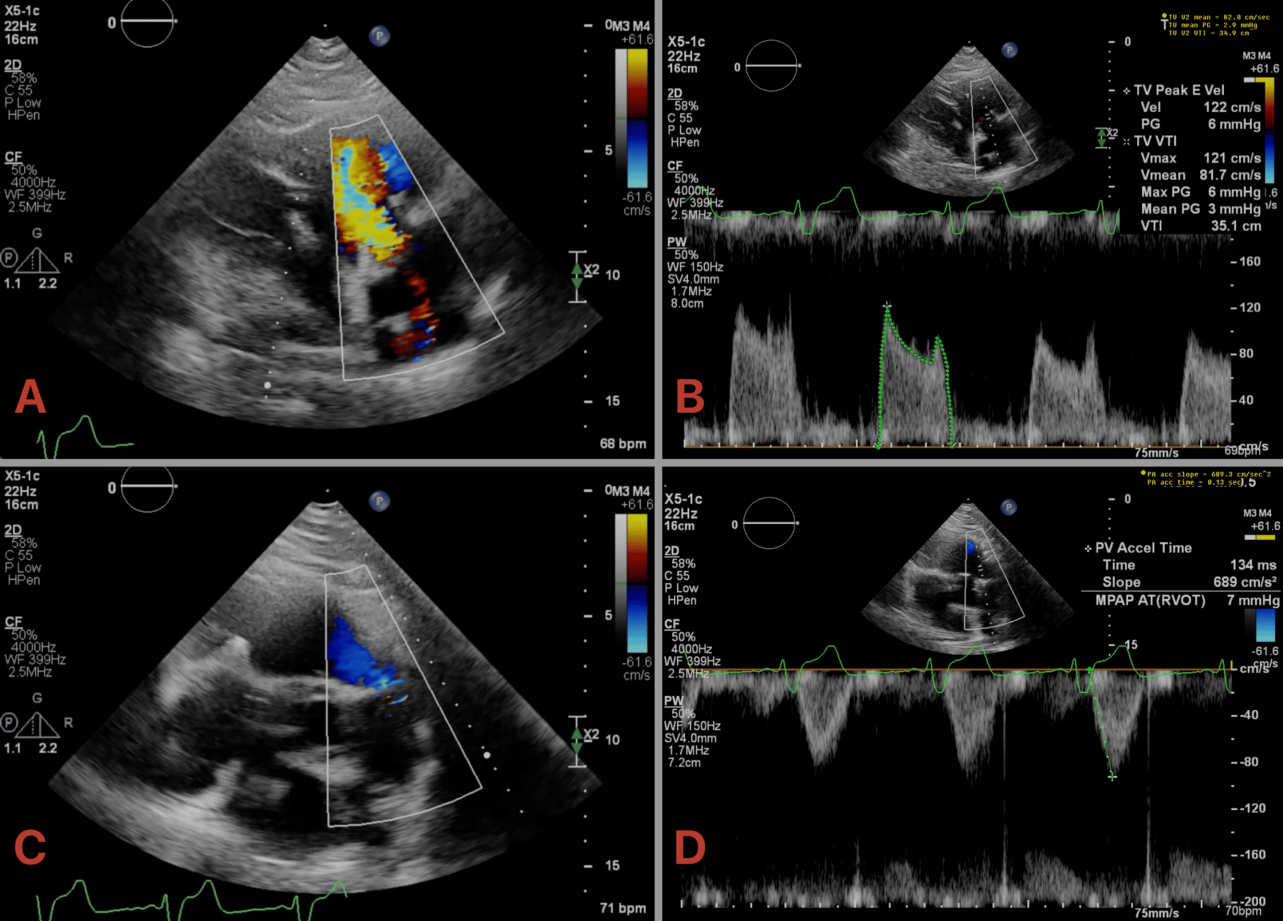
Postoperative transthoracic echocardiography (TTE) demonstrating well-functioning bioprosthesis following dual right-sided valve replacement TTE demonstrating (A) well-functioning bioprosthetic tricuspid valve (TV) with (B) normal gradients (3 mmHg) and (C) normally functioning bioprosthetic pulmonic valve (PV) with (D) normal gradient (7 mmHg).

The patient recovered well postoperatively with complete resolution of his right-sided heart failure. Follow-up TTEs have demonstrated well-functioning valves with normal gradients (Figure 3A-3D, Videos 5, 6). A DOTATATE PET scan demonstrated minimal disease in the liver. He was continued on medical therapy with lanreotide with close surveillance as per oncology and cardiology. Multidisciplinary management, including valve replacement and tumor debulking, led to the resolution of symptoms and normalization of right ventricular systolic function. Postoperative imaging showed reduced RV dilation with normal RV systolic performance.

**Video 5:** Postoperative transthoracic echocardiography (TTE) demonstrating well-functioning bioprosthetic tricuspid valve (TV)

**Video 6:** Postoperative transthoracic echocardiography (TTE) demonstrating well-functioning bioprosthetic pulmonic valve (PV)

## Discussion

CHD is a rare but serious complication of metastatic NETs, predominantly affecting the right-sided heart valves. It occurs due to excessive secretion of vasoactive substances, such as serotonin, by metastatic tumors, leading to endocardial fibrosis and valvular dysfunction [1]. The pathophysiology of CHD is primarily linked to prolonged exposure to these vasoactive agents, which cause progressive thickening and retraction of the valvular leaflets, ultimately resulting in tricuspid and pulmonary valve regurgitation. The valvular fibrosis observed in CHD is a direct consequence of persistent serotonin exposure, which is largely deactivated in the lungs. However, in patients with liver metastases, this detoxification process is bypassed, leading to excessive serotonin reaching the right-sided heart structures [2]. In some cases, right-sided heart failure ensues, significantly impacting morbidity and mortality.

Epidemiologically, CHD is reported in 50-60% of patients with carcinoid syndrome, often indicating advanced disease [3]. The clinical manifestations are diverse, including dyspnea, fatigue, peripheral edema, and ascites, which are often mistaken for other cardiac or systemic conditions. Given the potential for delayed diagnosis and progression to severe cardiac dysfunction, early recognition and a multidisciplinary approach are crucial for optimal management.

The choice of surgical management in these patients is complex and necessitates a collaborative effort across multiple specialties. Right-sided valve replacement has been shown to improve functional outcomes and survival, particularly when performed before the onset of severe right heart failure [4]. The decision to use bioprosthetic valves in our patient was based on the expected need for future oncologic surgeries and the risk of thrombosis associated with mechanical valves in patients with high serotonin levels [5].

Despite successful surgical intervention, CHD remains a progressive condition that requires continued oncologic and cardiac surveillance. Medical therapy with somatostatin analogs plays a crucial role in symptom control and slowing disease progression. Additionally, liver-directed therapies, such as transarterial embolization or peptide receptor radionuclide therapy (PRRT), may be considered in patients with significant tumor burden [6].

This case underscores the importance of early recognition and a comprehensive, multidisciplinary approach to the management of CHD. Future studies should explore novel therapeutic targets and optimal timing for surgical intervention to further improve outcomes in this patient population.

## Conclusions

This case highlights the successful management of severe CHD through a combination of medical therapy, perioperative hormonal management, and surgical intervention. The use of a multidisciplinary approach ensured an optimal balance between cardiac symptom control and oncologic treatment planning. Ongoing follow-up and disease surveillance remain essential to address potential tumor progression and future cardiac complications.
